# Evolution of flower allometry and pigmentation in *Mammillaria haageana* (Cactaceae)

**DOI:** 10.1186/s12870-021-03386-8

**Published:** 2022-01-25

**Authors:** Ulises Rosas, Elisa Sofía Fuentes-Pérez, Cristian R. Cervantes, Estela Sandoval-Zapotitla, Itzel Santiago-Sandoval, Salvador Arias, Jerónimo Reyes-Santiago

**Affiliations:** 1 Jardín Botánico, Instituto de Biología, Universidad Nacional Autónoma de México, 04510 Mexico City, Mexico; 2 Posgrado en Ciencias Biológicas, Universidad Nacional Autónoma de Mexico, Mexico City, Mexico

**Keywords:** Natural variation, Flower evolution, Cacti, Morphometrics, Cactoideae, Pigmentation, Tepal, Perianth

## Abstract

**Background:**

A puzzle in evolution is the understanding of how the environment might drive subtle phenotypic variation, and whether this variation is adaptive. Under the neutral evolutionary theory, subtle phenotypes are almost neutral with little adaptive value. To test this idea, we studied the infraspecific variation in flower shape and color in *Mammillaria haageana*, a species with a wide geographical distribution and phenotypic variation, which populations are often recognized as infraspecific taxa.

**Results:**

We collected samples from wild populations, kept them in the greenhouse for at least one reproductive year, and collected newly formed flowers. Our first objective was to characterize tepal natural variation in *M. haageana* through geometric morphometric and multivariate pigmentation analyses. We used landmark-based morphometrics to quantify the trends of shape variation and tepal color-patterns in 20 *M. haageana* accessions, belonging to five subspecies, plus 8 *M. albilanata* accessions for comparison as the sister species. We obtained eight geometric morphometric traits for tepal shape and color-patterns. We found broad variation in these traits between accessions belonging to the same subspecies, without taxonomic congruence with those infraspecific units. Also the phenetic cluster analysis showed different grouping patterns among accessions. When we correlated these phenotypes to the environment, we also found that solar radiation might explain the variation in tepal shape and color, suggesting that subtle variation in flower phenotypes might be adaptive. Finally we present anatomical sections in *M. haageana* subsp. *san-angelensis* to propose some of the underlying tepal structural features that may give rise to tepal variation.

**Conclusions:**

Our geometric morphometric approach of flower shape and color allowed us to identify the main trends of variation in each accession and putative subspecies, but also allowed us to correlate these variation to the environment, and propose anatomical mechanisms underlying this diversity of flower phenotypes.

**Supplementary Information:**

The online version contains supplementary material available at 10.1186/s12870-021-03386-8.

## Introduction

The nature of phenotypic variation is a problem that perplexed generations of scientists, including Darwin himself. Flower evolution has been adopted as a model to understand these bases of natural variation [1]. On the one hand, when discrete variation has been observed, for instance in flower color, it has been possible to associate it to the underlying biochemical pathways [2], or to selective genetic sweeps [3], or variation driven by the environment [4]. However, when the phenotypic variation is subtle, identifying its underlying bases is rather a difficult task. Based on what has been observed in other model organisms (i.e. yeast) [5], whether the variation is subtle or discrete, one can predict that the whole breath of phenotypic variation is mainly adaptive but this assumption is contingent to the specifics of the measured phenotypes. In other words, not all quantified phenotypes might be important for natural selection, and not all phenotypes under natural selection have been quantified. Thus, when studying subtle variation in naturally occurring variants, robust and unbiased phenotyping methods are ideal.

The raise of geometric morphometric methods has aided the less biased characterization of organs and organisms based on predetermined landmarks, from which the main trends of variation are extracted. Thanks to them, complex phenotypes have been quantified and even genetic bases underlying them have been identified for other organs [6], as well as flowers [7, 8]. Here we use geometric morphometric approaches to quantify floral attributes in the highly complex floral organ of cacti.

In cacti, flowers are described as specialized buds embedded in the tissue of a stem. That is, the ovary is surrounded by material derived from stem or receptacle tissue, forming a structure called pericarpel. The other structure that makes up the flower is the perianth, composed of sepals and petals that develop in a spiral arranged with a gradual transition from green bracts to pigmented structures [9]. The set of both whorls that cannot be distinguished within the first and second whorls are called tepals [10]. Typically, the genus *Mammillaria* Haw. has a particular type of perianth: flowers are infundibuliform and have a short perianth and a bare pericarpel [11]. In particular, the *Mammillaria haageana* Pfeiff. flower has a long receptacle tube, the outer tepals are linear-lanceolate with pink to purple pigmentation, and the inner tepals are pink to purple with a darker pink middle stripe [12].

In a previous study, we tested the monophyly of the Series *Supertexta* in *Mammillaria*, a group that estimate to have diverged from its sister Series (*Polyacanthae*), approximately 2.1 Ma ago [13]. Within the Series *Supertextae*, one of the accepted classifications [14] recognizes nine species, which include: *M. albilanata* Backeb., *M. crucigera* Mart., *M. columbiana* Salm-Dyck, *M. dixanthocentron* Backeb., *M. flavicentra* Backeb., *M. haageana*, *M. halbingeri* Boed., *M. huitzilopochtli* D.R.Hunt, and *M. supertexta* Mart. Ex Pfeiff.. Within *M. haageana*, six subspecies have been recognized [15], which include the subspecies: *M. haageana* subsp. *haageana*, *M. haageana* subsp. *acultzingensis* (Linzen et al.) D.R.Hunt, *M. haageana* subsp. *conspicua* (J.A.Purpus) D.R.Hunt, *M. haageana* subsp. *elegans* D.R.Hunt, *M. haageana* subsp. *meissneri* (C.Ehrenb.) U.Guzmán, *M. haageana* subsp. *san-angelensis* (Sánchez-Mej.) D.R.Hunt, and *M. haageana* subsp. *vaupelii* (Tiegel) U.Guzmán. Among these species, we found that *M. haageana* units have a convoluted evolutionary history, that might involve *M. albilanata* evolutionary units [13]. This is the reason why we decided to identify patterns of floral variation in *M. haageana* and include *M. albilanata* as a group for comparisons.

**Fig. 1:**
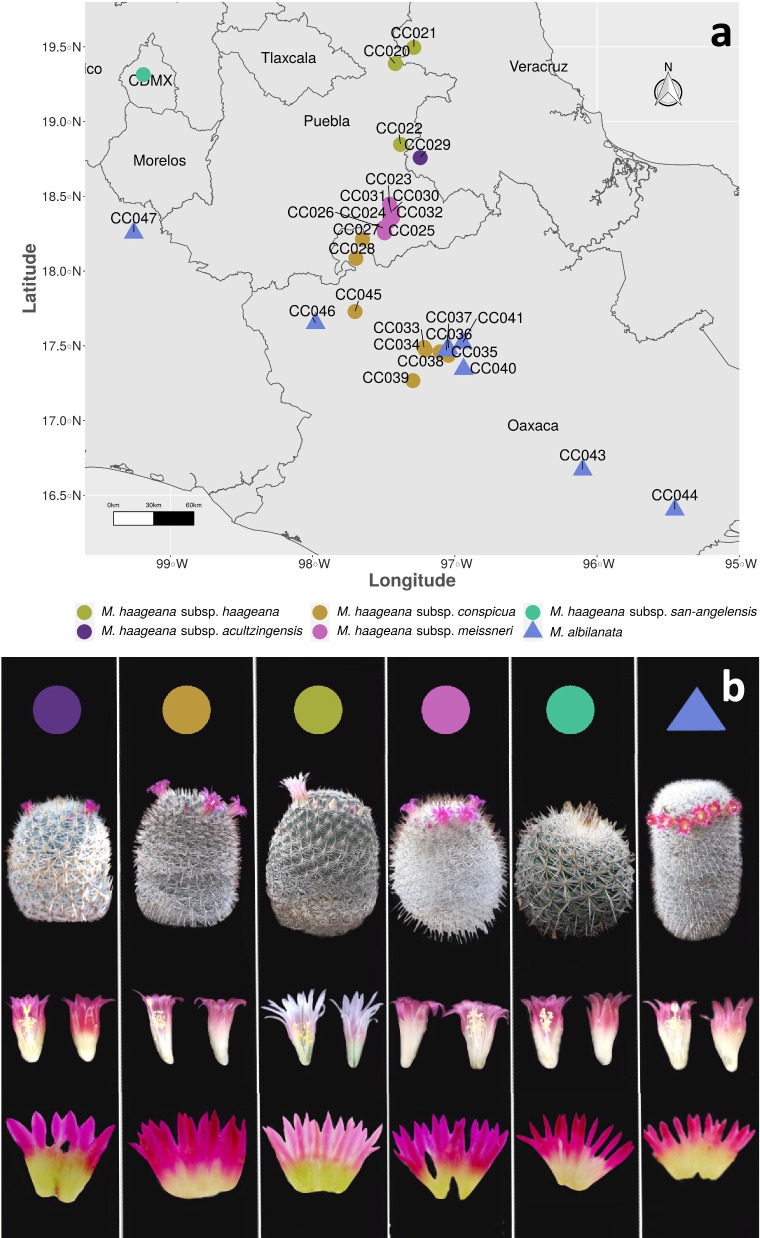
*Mammillaria haageana* (and its sister species *M. albilanata*) distribution and phenotypes, color-coded according to the previously assigned subspecies. **a** Distribution of collected plant materials. **b** Plant and flower phenotype variation

Thus, in this study, geometric morphometrics is used as a method to quantify complex flower phenotypes, and we test whether there is a link between subtle phenotype evolution and the environment within a cacti species (Fig. 1). Secondly, we evaluated taxonomic relationships within the species *Mammillaria haageana.* We also hypothesize that the morphogeometric variation patterns of the perianth of *M. haageana* support the grouping of infraspecific taxa of the current classification [15]. Therefore, this study aims to characterize, through morphogeometric analysis, the natural variation of the perianth of *M. haageana*. With this, we aimed at identifying what environmental factors might be driving trait evolution in the *Mammillaria haageana* perianth, as well as the understanding of patterns of morphogeometric variation that might allow the clarification in the classification of infraspecific taxa within the species. Finally, we performed anatomical studies of perianth sections in *M. haageana* subsp. *san-angelensis* to propose some underlying structural features of the tepal that can provide information about the tepal evolution.

## Results

### Four shape principal components describe tepal variation in *M. haageana* and its sister species

**Fig. 2:**
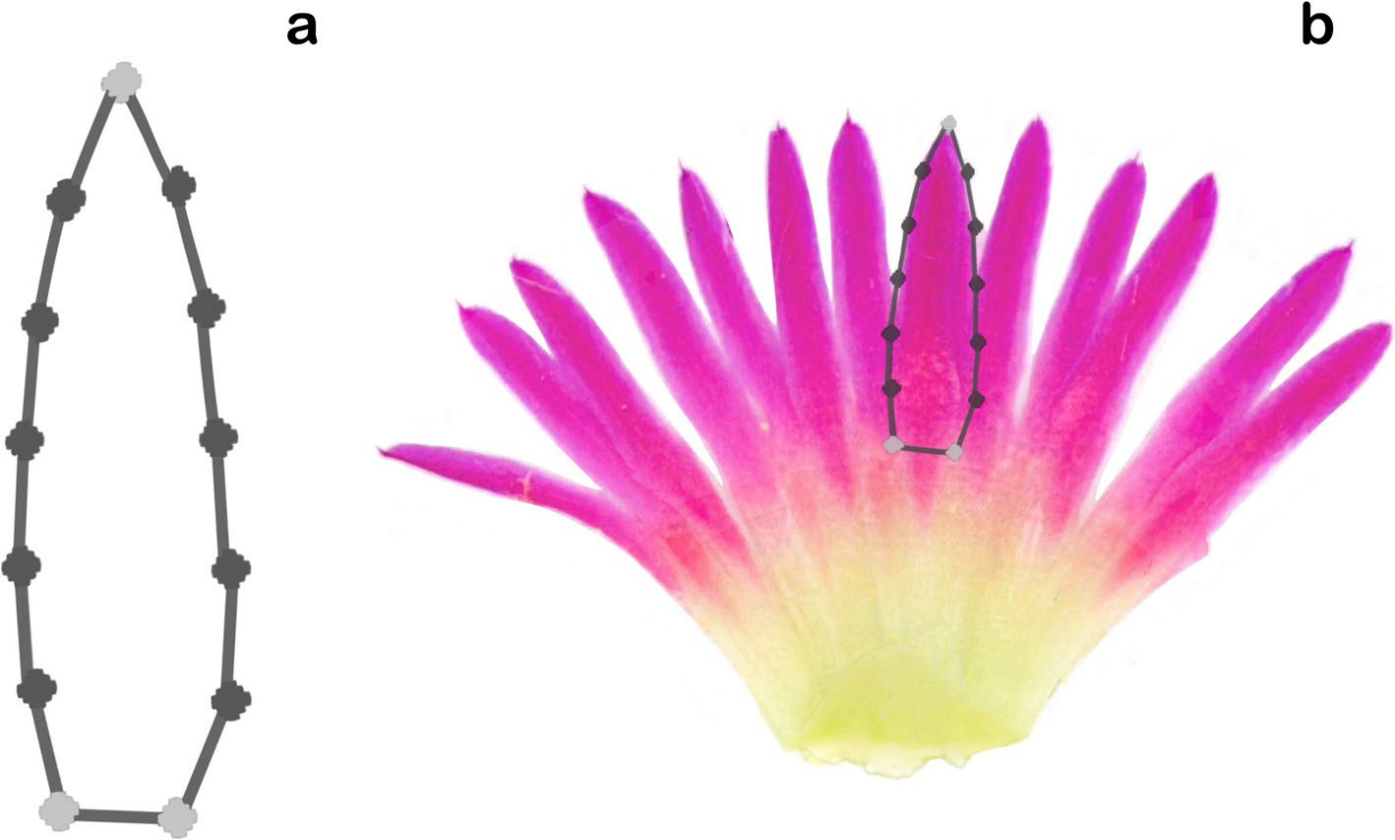
Template used for the point model in the morphometric analyses. **a** 13-point model template, including primary landmarks (grey dots), and semi-landmarks landmarks (black dots). **b** Point-model template overlaid on a tepal from a flattened flower

We characterized shape and color-pattern variation in a collection of 20 *M. haageana* accessions that belonged to the six subspecies as previously proposed [15], plus eight accessions from the sister species *M. albilanata*, giving a total of 717 tepals, from 272 flowers, from 28 accessions. We have recently used some of these accessions to characterize the natural variation in root architecture development [16]. To perform the tepal shape analysis, we flattened the perianth and fitted a 13-landmark template on each studied tepal (Fig. 2). First, to extract the tepal shape variation, we performed a Principal Component Analysis (PCA) with Procrustes for rotation, translation, but not size, because size is one of the features that display variation among the *M. haageana* accessions.

**Fig. 3:**
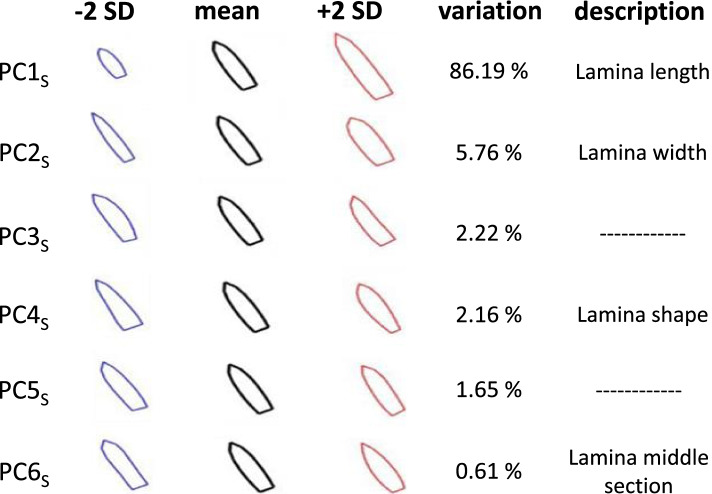
Tepal shape and size is described by at least six Principal Components (PC_S_). The deviations from the mean shape (black outline) are represented in blue (− 2 standard deviations) or in red (+ 2 standard deviations). The percentage of explained variance is shown, together with our description of the corresponding PC_S_ trend

We found that 99% of the variation was covered by eight Principal Components (PCs), but the 7th and 8th capture less than 0.1% of the variation in each, and their variation was so subtle that could not be described. The first 6 PCs captured 98.59% of the variation in tepal shape (Fig. 3). The first shape Principal Component (PC1_S_) a large proportion of the variation (86.19%) confirming that *M. haageana* displays wide phenotypic variation in tepal size. Accordingly, PC1_S_ seems to capture the tepal lamina length. PC2_S_ is an orthogonal axis to PC1_S_, and therefore should capture the shape, regardless of the size; thus PC2_S_ captured 5.76% of the variation and seems to quantify the tepal lamina width. PC3_S_ captured 2.22% of the variation, and seems to capture whether the tepal is tilted towards the left or the right; however we believe this to be an artefact of the tepal flattening process, and therefore it was not taken into account. PC5_S_ captured 2.16% of the variation, which seems to capture aspects of the tepal lamina shape, such as the width of the proximal end, or the sharpness of the distal end. PC5_S_ captures 1.65% of the variation but similar to PC3_S_, we believe this to be an artefact of the teal flattening process, and thus it was not further considered. PC6_S_ captures 0.61% of the variation and seems to describe the width of the middle section of the lamina. These four shape Principal Components (PC1_S_, PC2_S_, PC4_S_ and PC6_S_) were then considered to evaluate shape variation in *M. haageana* accessions, plus its sister species *M. albilanata.*

**Fig. 4:**
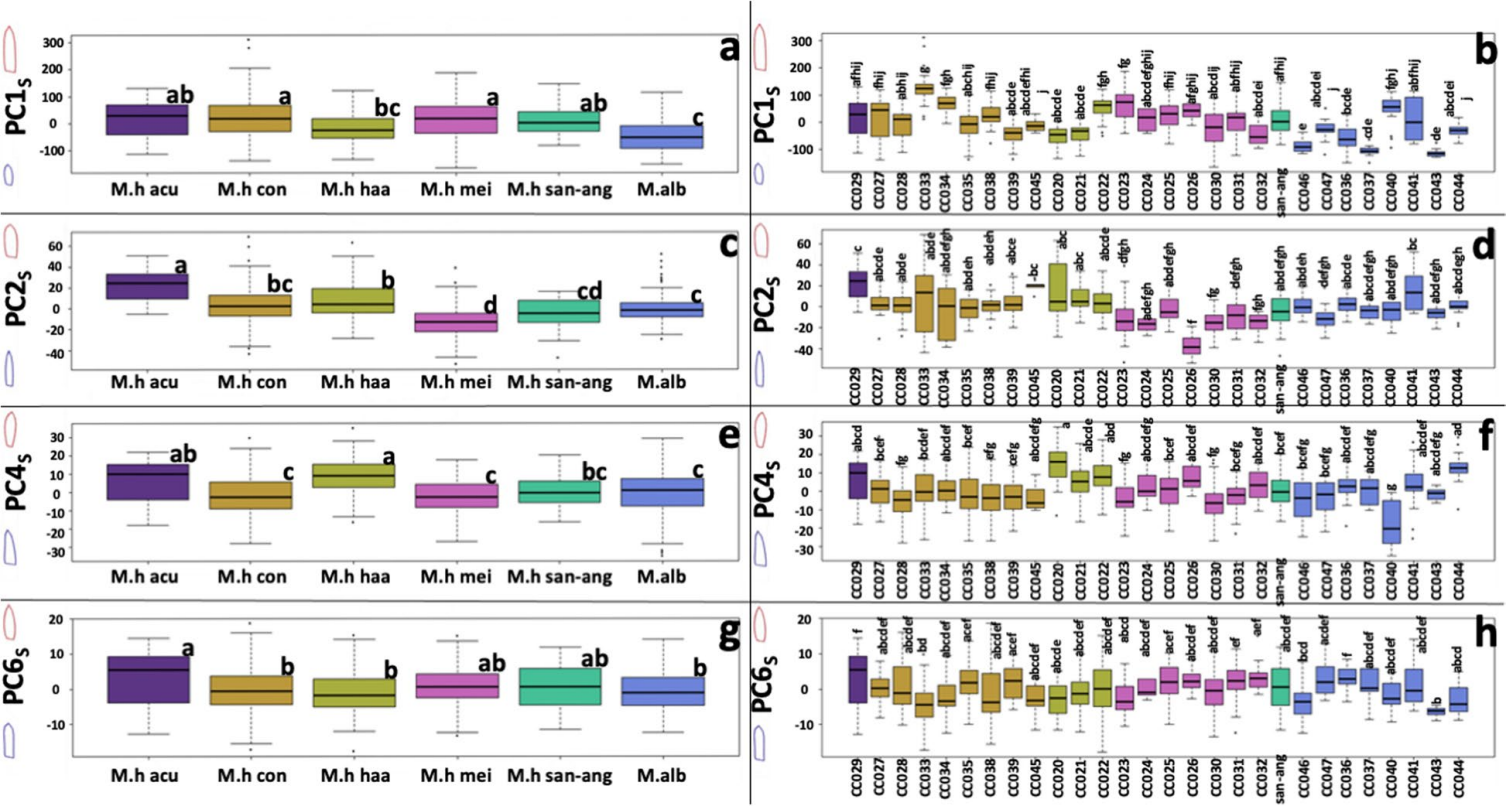
Tepal shape and size variation according to the PC_S_ axes. **a,c,e,g** depicts the PC_S_ groups according to the previously proposed subspecies. **b,d,f,h** depicts the PC_S_ groups according to their corresponding accession. Boxplots show the Q1, Q2, and Q3 quantiles, and outliers. Statistically homogeneous groups from a Dunn test (*p* < 0.05) are also depicted with letters

We then plotted the biologically meaningful shape PCs according to their subspecies or their accession (Fig. 4). We noted that according to PC1_S_, *M. albilanata* has the smallest tepals, while *M. haageana* subsp. *conspicua* and *M. haageana* subsp. *meissneri* had the largest tepals (Fig. 4a). Concerning the lamina width regardless of size (PC2_S_), *M. haageana* subsp. *acultzingensis* had the widest tepals, while *M. haageana* subsp. *meissneri* had the narrowest tepals (Fig. 4c). On lamina shape (PC4_S_) *M. haageana* subsp. *haageana* had the widest distal end of the tepal, but there were no differences between *M. albilanata* as compared to three other *M. haageana* subspecies (Fig. 4e). On the lamina middle section (PC6_S_), the subspecies had broad variation and little distinction was observed between them (Fig. 4g). Despite the variations observed between subspecies, differences between accessions belonging to the same subspecies were far more striking. For instance, on PC1_S_ we observed ample variation within the *M. haageana* subsp. *conspicua* accessions, or *M. haageana* subsp. *haageana* accessions; but a similar pattern was observed within the *M. albilanata* accessions (Fig. 4b). This pattern was also observed in the other shape traits PC2_S_, PC4_S_, and PC6_S_, which was less obvious in the later. In other words, each accession has a distinctive set of shape traits, which seemingly do not agree with their assigned subspecies.

### Four color principal components describe tepal variation in *M. haageana* and its sister species

**Fig. 5:**
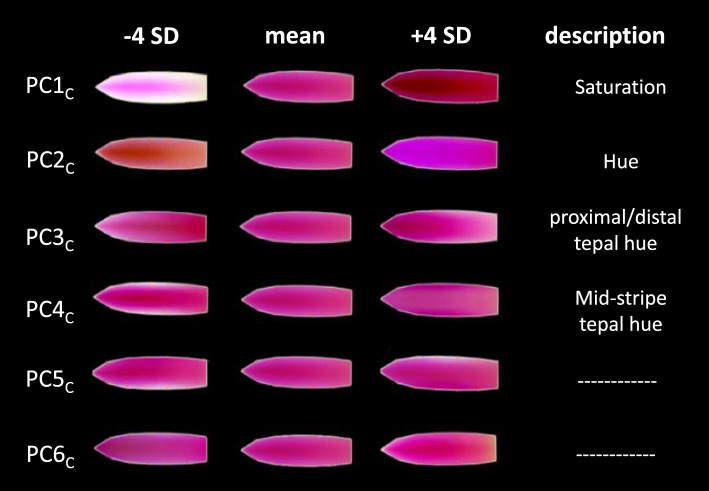
Tepal color is described by at least six Principal Components (PC_C_). The deviations from the mean shape are represented to the left (− 4 standard deviations) or to the right (+ 4 standard deviations). The description of the PC_C_ trend is shown

Using the geometric morphometric landmark data-points, we warped all the tepal images into the mean shape, using 10,000 pixels contained in the polygon (Fig. 2) in the RGB channels (30,000 pixels). With this new dataset, we performed a PCA to extract the main trends of variation in tepal color. 95% of the variation was captured by 47 color PCs. We only present the first 6 PCs, as they seem to capture trends of variation easily be visualized (Fig. 5). Other PCs have very subtle trends that could not be described (data not shown). The first color Principal Component (PC1_C_) seems to capture the color saturation; the PC2_C_ captures the hue of the tepal; PC3_C_ captures the proximal-distal hue of the tepal, and PC4_C_ captures the mid-stripe hue. PC5_C_ seems to capture left-right hue, which we believe to be an artefact because the right or left-handedness of the pigmentation is not observed in *M. haageana* natural accessions. PC6_C_ was also not described and further considered, as the variation it captures is very subtle. Thus, we propose four color geometric morphometric traits (PC1_C_, PC2_C_, PC3_C_ and PC4_C_) in *M. haageana* (and *M. albilanata*) to compare pigmentation patterns between and within subspecies.

**Fig. 6:**
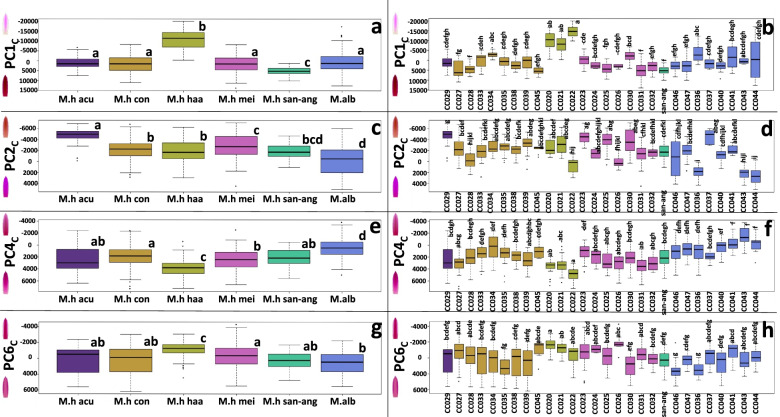
Tepal color variation according to the PC_C_ axes. **a,c,e,g** depicts the PC_C_ groups according to the previously proposed subspecies. **b,d,f,h** depicts the PC_C_ groups according to their corresponding accession. Boxplots show the Q1, Q2, and Q3 quantiles, and outliers. Statistically homogeneous groups from a Dunn-test are also depicted with letters

Similarly to what we did on the shape, we plotted the biologically meaningful color PC_C_ according to their subspecies (Fig. 6). Regarding saturation (PC1_C_), we found little variation between most *M. haageana* subspecies, except for *M. haageana* subsp. *haageana* which has lighter saturation tones; on the other hand, *M. haageana* subsp*. san-angelensis* has darker saturation tones (Fig. 6a). Concerning hue (PC2_C_), *M. haageana* subsp. *acultzingensis* had more reddish tepal color as compared to the rest of the *M. haageana* subspecies, but *M. albilanata* had the rather magenta tepal colors (Fig. 6c). On the proximal-distal tepal hue (PC4_C_), and similar to the distinctive patterns observed in PC1_C_, *M. haageana* subsp. *haageana* has darker magenta pigmentation towards the apex, whereas *M. albilanata* has a darker magenta color towards the proximal end of the tepal (Fig. 6e). In other words, *M. haageana* subsp. *haageana* and *M. albilanata* share pigmentation patterns, but not with the complement of subspecies recognized in *M. haageana*, based on PC1_C_ and PC4_C_. Finally, the mid-stripe tepal hue (PC6_C_) is more prominent and noticeable in *M. haageana* subsp. *haageana*, further supporting the distinction of this subspecies from the rest (Fig. 6g).

While differences in color-pattern variations were remarkable between subspecies, when comparing accessions only PC1_C_ and PC4_C_ showed broad differences between accessions, even from the same subspecies. This was the case of *M. haageana* subsp. *meissneri* on PC1_C_, in which CC031 has significantly lower PC1_C_ value (saturation) than CC030. Another example is the *A. albilanata* accession CC044 in which there is ample variation between individuals of the same accession, that is noticeable by the amplitude of the boxplot (Fig. 6b). Remarkably, *M. haageana* subsp. *haageana* accessions are similar to one another in PC1_C_ and PC4_C_.

### Phenetic analyzes suggests possible natural groups

**Fig. 7:**
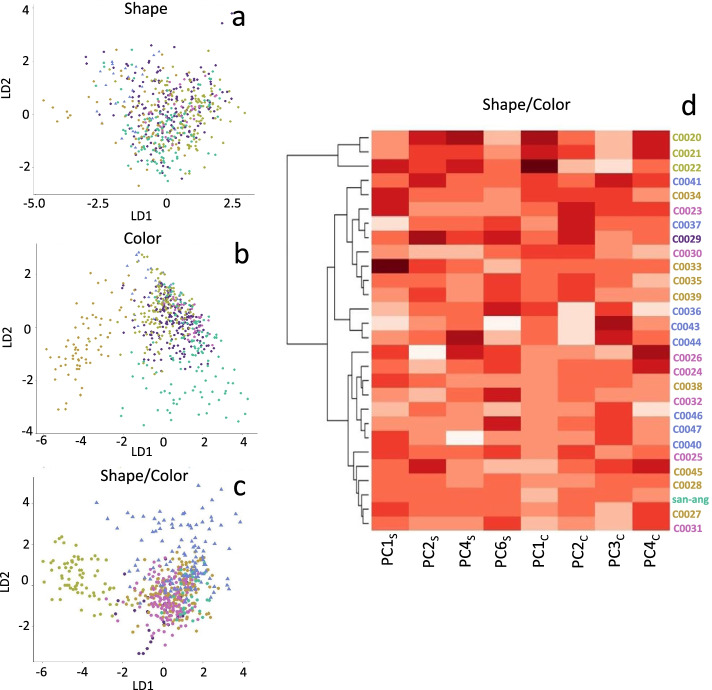
Multivariate analysis of flower phenotypes. **a** LDA of flower phenotypes using only PC_S_ (shape) values. **b** LDA of flower phenotypes using only PC_C_ (color) values. **c** LDA of flower phenotypes using both PC_S_ (shape) and PC_C_ (color) values. **d** Euclidean distance clustering and heat map using both PC_S_ (shape) and PC_C_ (color) values

Both shape and pigmentation analyses provided quantitative parameters of novel axes to compare the accessions of *M. haageana* and its sister species *M. albilanata*. As shown in Fig. 4 and Fig. 6, some traits partially recapitulate subspecies classifications, but others do not. To unravel the possibility that these traits could recapitulate the subspecies classification, a series of multivariate analyses were performed. First, a Linear Discriminant Analysis (LDA) with shape traits was implemented to test whether the four PC_S_ parameters separated the five subspecies plus the sister species (Fig. 7a). This model gave a precision of 65.45% in the correct assignment of accessions. In other words, in an LDA, the four shape components in *M. haageana* failed to create a distinction between the five subspecies and the sister species. Subsequently, the four parameters corresponding to pigmentation were tested (PCc). The model gave a 80.67% accuracy in assigning the accessions to the subspecies. Nevertheless, in this analysis it was possible to see the separation of two groups of accessions corresponding to *M. haageana* subsp. *conspicua* and *M. haageana* subsp. *san-angelensis* (Fig. 7b). Finally, we performed a third LDA combining the eight components corresponding to shape (PC_S_) and color (PC_C_). This model has an accuracy of 71.32% in assigning subspecies. In this case, the two groups of accessions that separated from the rest were the species *M. albilanata* and the subspecies *M. haageana* subsp. *haageana* (Fig. 7c). In summary the LDA of shape and pigmentation parameters only partially recapitulated some of the previously proposed taxonomic units [15].

We also used another multivariate method based on Euclidean distances to test whether the taxonomic units can be recapitulated. This gave groupings between the accessions based on shape and color. It was observed that the accessions CC020, CC021 and CC022, corresponding to the subspecies *M. haageana* subsp. *haageana*, are grouped in a single branch as compared to the rest of the accessions (Fig. 7d). This result might indicate that the combination of shape and color parameters in *M. haageana* subsp*. haageana* could indeed define this group as a subspecies within *M. haageana*.

**Fig. 8:**
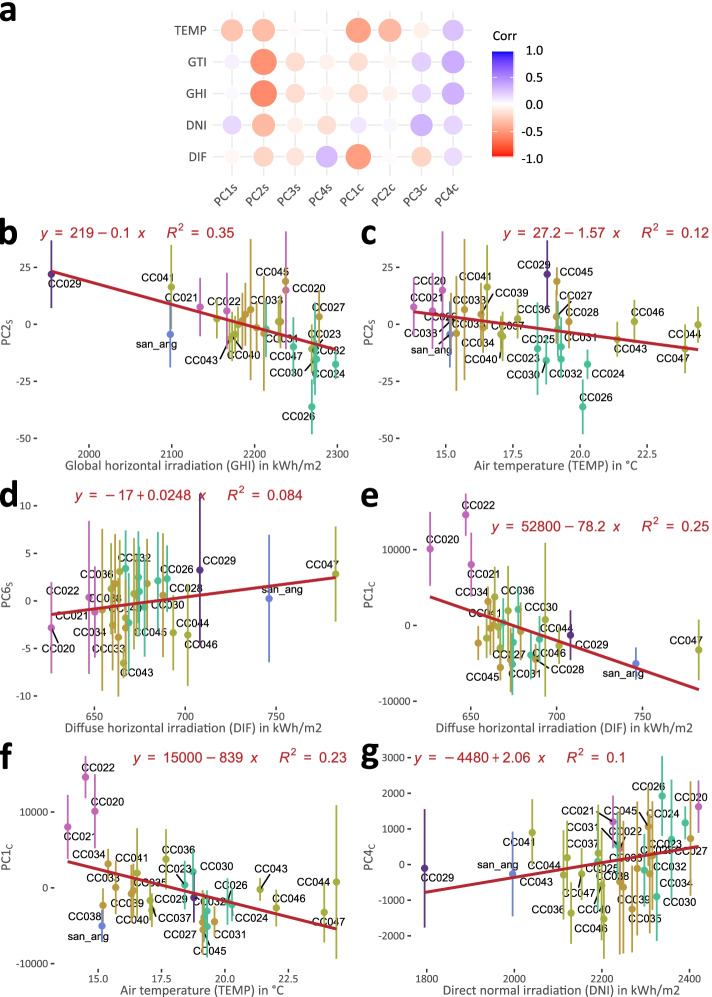
Tepal shape and color are correlated to environmental variables. **a** Solar radiation environmental variables and their correlation to shape (PC_S_) and color (PC_C_). **b-d** Top correlations of solar radiation variables to tepal shape (PC_S_). **e-g** Top correlations of solar radiation variables to tepal color (PC_C_). GHI, long-term yearly average Global Horizontal Irradiation; DNI, long-term yearly average of Direct Normal Irradiation; DIF, long-term yearly average of Diffuse Horizontal Irradiation; GTI, long-term yearly average of Global Irradiation at Optimum Tilt (kWh/m^2^); and TEMP, long-term yearly average of air Temperature (°C). Accessions are color-coded according to the previous subspecies classification. Dots represent the mean PC value for each accession, and lines represent the standard deviation from the mean

Pigmentation patterns are known to have impacts on color reflectance, and therefore color perception by pollinators, as demonstrated in *Antirrhinum* [17]. In order to test whether the environment might play a role in determining tepal pigmentation patterns in *M. haageana*, we performed an association analysis of solar radiation with our shape and color PCs (Fig. 8a). Interestingly, we found several correlations between solar radiation, with tepal shape and color. For simplicity, we show the plots of the six highest correlations between the environment and tepal attributes (Fig. 8b-g). Regarding tepal shape, we found that global horizontal radiation (GHI) and air temperature (TEMP) were negatively correlated with PC2_S_ (lamina width) (Fig. 8b-c), implying that narrower tepal accession originates from cooler locations with lower global horizontal irradiation, while wider tepal accessions originated from warmer and higher global horizontal irradiation locations. We also found a positive correlation between the organ shape parameter PC6_S_ (lamina middle section) and global horizontal radiation (GHI) (Fig. 8d), which interpretation requires further examination. Regarding tepal color, we found that PC1_C_ (saturation) has a negative correlation with diffuse horizontal radiation (DIF) (Fig. 8e); this means that darker tepals are present in accessions originally from locations with lower DIF, while lighter tepals are present in accessions originally from locations with higher DIF. Another interesting negative correlation was found between PC1_C_ (saturation) and air temperature (TEMP) (Fig. 8f), in which paler tepals (low PC1_C_) were from accessions originally from lower TEMP locations, as opposed to darker tepals (high PC1_C_) which were from accessions from higher TEMP locations. On the other hand, PC4_C_ (mid-stripe tepal hue) was positively correlated with direct normal radiation (DNI) (Fig. 8g), in which the more noticeable mid-stripe (low PC4_C_), the lower DNI; meanwhile the less noticeable mid-stripe (high PC4_C_) the higher DNI. Overall, these results allowed us to establish a link between tepal shape and pigmentation patterns, and the environment, which might definitely have adaptive bases.

**Fig. 9:**
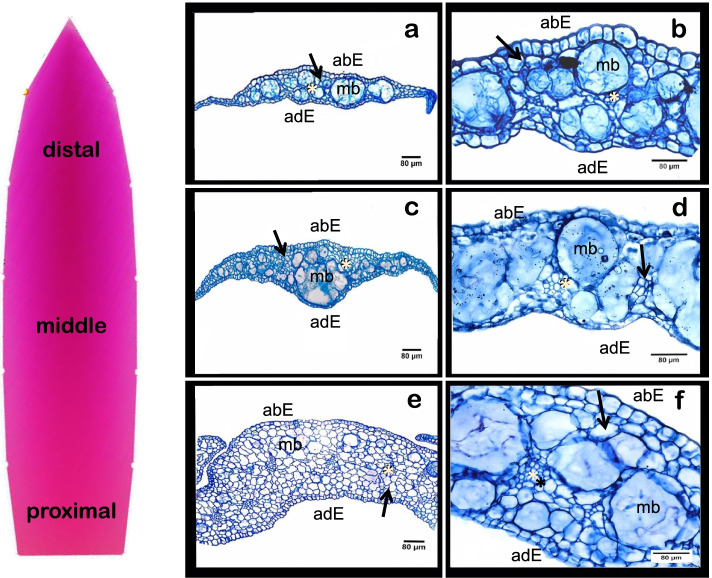
Cross-section of in *Mammillaria haageana* subsp. *san-angelensis* along different longitudinal regions. **a-b** distal section, **c-d** middle section, and **e-f** proximal section. A modeled tepal is shown on the left-hand side for reference. abE abaxial epidermis, adE adaxial epidermis, mb mucilage body, light asterisk indicates vascular bundles, and black arrows represent mesophyll cells

In order to provide a possible anatomical explanation on the origins of shape and color variation in *M. haageana* tepals, we examined the tepal anatomy of *M. haageana* subsp. *san-angelensis* (Fig. 9). To do this we performed histological sections of the proximal, middle, and distal sections of the tepals, and characterized the tepals qualitatively and quantitatively. We obtained that epidermis of the abaxial and adaxial sides are fairly similar to each other, and also similar cell size at the distal or proximal end of the tepal. These sections suggest that color variation along the transversal and longitudinal sections of the tepal lamina does not originates within the epidermis cell shape, and possibly the variation between accessions either. Thus, the variation in tepal color that we characterized between accessions must be given by the variation in betalain (purple color) and betaxanthin (yellow color) pigments, which concentrations remain to be quantified. Last, within the mesophyll we found large mucilage bodies, which sometimes seemed to be large single cells, but in some other cases, these mucilage bodies were surrounded by small cells. These mucilage bodies had a variety of sizes and occupied most of the mesophyll tissue towards the distal part of the tepal and were rather small at the proximal end of the tepal. On the other hand, the tepal was highly vascularized towards the proximal end, but fewer or smaller vascular bundles were observed towards the distal end. In leaves, vascular bundles are more prominent towards the leaf base, and at the apex they become mere vascular endings with tracheal elements. In the context of cacti tepals, this inverse relationship of vasculature versus mucilage bodies in the mesophyll might be part of the tepal adaptation for the flower to avoid desiccation towards the distal end, but maintain water flow towards the proximal end. Finally, to contextualize what we observed in *M. haageana* subsp. *san-angelensis*, we found that tepal anatomy is fairly similar to what has been observed in tepals of *Epiphyllum phyllanthus* [18], another distantly related cacti species, suggesting that tepal anatomy is fairly constant in the Subfamily Cactoideae.

## Discussion

In this work we studied the natural variation of flower shape and color between subspecies and accessions of *M. haageana* and its closely related species *M. albilanata.* Using geometric morphometric methods, we established eight multivariate quantitative parameters to describe variation on several tepal features. When grouping together the tepals from each subspecies, we found little variation between the allometric phenotypes, however, when teasing apart the variation from each accession, we found a wide range of variation within each subspecies. One exception was *M. haageana* which in at least four allometric parameters were distinctive from the rest. The other exception was *M. albilanata* which in some other parameters were distinctive. Phenetic multivariate analyses using the geometric morphometric parameters showed that the subspecies, except for *M. haageana* subsp. *haageana* and *M. albilanata*, show that accessions do not group together according to the subspecies they belong to. In fact, the accessions rather group according to global horizontal irradiation (GHI) and mainly, among other environmental factors. This can be interpreted in two ways: a) the evolution of shape and size in *M. haageana* is independent from subspecies divergence, or b) the current subspecies classification is not natural. Further studies are required to respond to this issue.

Pollinator occurrence can sometimes explain flower color variation within species, but in some other instances it is rather abiotic factors such as temperature and solar irradiation what underlies color variation, as was the case of *Campanula americana* (Campanulaceae) [19]. This is because solar irradiation indirectly affects the pollinator behavior by modifying floral temperature, or color perception. Nevertheless, it has also been found that floral color variation can also be explained by phenotypic plasticity. This was the case of the selfing species *Boechera stricta* (Brassicaceae), whose flower colors in natural populations ranges from mostly white, to pink and purple colors; it was found that floral pigmentation was higher when plants were studied in the field, as opposed to plants in greenhouse conditions; yet from the later experiments it was determine that flower color is highly heritable when comparing populations [4]. The authors proposed that increased flower pigmentation is correlated to biotic and abiotic stresses in natural conditions. This poses the possibility that *M. haageana* flower shape and color variation might be a byproduct of solar radiation on pollination behavior, or that this variation might give fitness benefits under the wide range of environments where the accessions originate from.

Resolving the phylogenetic relationships between and within species of the *Mammillaria* genus has been a challenging puzzle for cacti taxonomists. Here we addressed the problem of floral attribute variation in *M. haageana*, taking into account several accessions belonging to subspecies according to previous classifications [15]. Yet, in most cases our tepal shape and color analyses did not manage to recover the proposed infraspecific categories. Some possible explanations are that: a) the infraspecific classification is natural, but *M. haageana* flowers have evolved independently from the diagnostic characters that were used to establish the subspecies, or b) an update on the infraspecific categories is required, taking into account not only floral characters, but vegetative and genomic evidences. Nevertheless, our multivariate analyses clearly identify some of the previously proposed taxa, such as *M. haageana* subspecies *haageana*, suggesting that this group might indeed be natural.

Ample evidence has demonstrated the close relationships between pollinators and the reproductive success of several cacti species [20–23]. In this regard, in other model species such as *Antirrhinum* (Plantaginaceae), epidermal cell shape could modify the color and perception of pollinators [17]. Although we only studied one accession (*M. haageana* subsp. *san-angelensis*) and pigmentation patterns vary along the transversal axis of the tepal, we did not find evidence of epidermis cell shape variation along the transversal or longitudinal sections, possibly suggesting that natural variation in pigmentation patterns might be rather explained by the type of pigments contained within the tepals. Furthermore, given the proportional size of the mucilage bodies within the mesophyll of the tepals, it is possible that within these mucilage bodies, the pigments (in cacti mainly betalains and betaxanthins) are stored, as well as in the epidermal cells, or subepidermal cells as has been previously found in cacti stems [24]. Regarding this, cacti flowers are severely exposed to high irradiation and temperature, and the presence of abundant mucilage bodies in the mesophyll might be an important anatomical adaptation to cope with those challenging environments. Given our observed correlation of solar irradiation and tepal pigment and shape, it remains to be addressed whether mucilage bodies might be involved in the natural variation and connection to solar radiation that we observed in this study.

Overall, the ample distribution and variety of environments that *M. haageana* occupies, opens new avenues of research to understand local adaptations, how traits evolved, and the evolutionary history of these natural variants. However, as in any phenotype characterized in a common environment, away from the natural distribution of the species, it remains to be tested whether the phenotypes in the field are identical to the phenotypes in the wild; yet we have no evidence to think otherwise. This is because floral meristems in succulent plants obtain their resources from their storage parenchyma accumulated over the years, rather than the prevailing soil conditions in a particular reproductive year. This is why we believe that *M. haageana* could be a model system to address relevant questions on the microevolution of succulent plants.

## Materials and methods

### Plant material

Two species of the genus *Mammillaria* series *Supertextae* were used, *M. haageana* and its sister species *M. albilanata*. The material was obtained from the Jardín Botanico at Instituto de Biología, UNAM Cactaceae Collection, where only five subspecies of *Mammillaria haageana* out of the seven were previously collected from the field, and deposited as living material for research purposes [15, 16]. Therefore, we used *M. haageana* subsp. *haageana*, *M. haageana* subsp. *conspicua*, *M. haageana* subsp. *meissneri*, *M. haageana* subsp. *acultzingensis* and *M. haageana* subsp. *san-angelensis*, as identified by CCS and SA, the cacti taxonomists in our research team. In the case of *Mammillaria albilanata*, only two subspecies were included, *M. albilanata* subsp. *albilanata* and *M. albilanata* subsp. *oaxacana* were used as the sister species for comparison. The specimens were assigned a code according to the locality in which they were collected. For future reference, the set of specimens belonging to a locality were called accessions. This to distinguish them, as we still do not know whether they can be considered as populations. From each plant, four individuals were selected per accession. In some accessions there were only three available individuals. From each individual, we aimed to collect three flowers in anthesis from each selected plant, but in some cases, we could only collect one or two flowers. In other words, we aimed at collecting 12 flower samples per accession, but in some cases we had fewer (Additional file 1). This gave a total of 28 accessions and 239 flattened flowers (Additional dataset 1).

### Morphometric analysis of shape

To obtain images of flattened tepals, first we removed one of the two rings of tepals (the outer tepals because often they have bracteole features). Then the remaining perianth was flattened over paper sheets using double-sided tape (Double-sided adhesive tape, © Red Top (18 mm × 20 m)). Subsequently, all the sheets of paper with the flattened perianth were scanned at a resolution of 1200 dots per inch (DPI) using an Epson Perfection V550Photo scanner. In this way, two-dimensional images of the dorsal view of the perianth, and particularly the inner tepals were obtained. From each flattener flower image, we selected three tepals for geometric morphometric analyses, which involved 699 tepals in total (Fig. 2a).

To generate allometry models we placed reference points on the tepal images. In *Mammillaria*, some part of the tepal might be fused or emerge deep down from the pericarpel. However, to simplify our analysis, we only took into account the portion of the tepal that is not fused, the tepal lobe. Thus, a 13-point model template was designed to capture the tepal shape of *M. haageana*, and its sister species *M. albilanata*. The model template was made of two types of points: primary and secondary landmarks. The primary landmarks, depicted in Fig. 2a in light grey, were fixed to distinguishable features of the tepals, such as the left or right corner at the base of the tepal lobe, or the tip of the tepal. The base of the tepal lobe was designated as the lowest point where two tepals are not fused, and tepals are pigmented. The secondary points, depicted in Fig. 2a in dark grey, were placed along the outline of the tepal, in such a way that they were evenly spaced between the primary landmarks. The point model was designed with the shape model AAMToolbox program [25, 26] implemented in the Matlab environment (Version 7.12.0.635).

To obtain size and shape variables from Cartesian coordinates, Procrustes Analysis (PA) was implemented in all images. The PA is a tool that allows comparing two homologous point configurations from two variants of the same entity. The first transformation operation was the translation, based on the displacement of all the points at a fixed distance in a certain direction. The second transformation operation was the rotation operation, which consists of moving all the points using a fixed angle around an axis. The third often used transformation, the scaling method, or uniform change of scale, was not applied within the study. This was done to take into account the size variation as an important factor to quantify, in addition to the shape. The analysis was carried out with capture of 99% of the variation in the values along the length, width, and curvature of the tepals in the different accessions. Once the shapes were aligned with the PA, a Principal Component Analysis was performed to determine the possible variations in the shape of the tepals.

### Morphometric analysis of color

The same specifications of the PA were applied for the Analysis of Principal Components of Coloring (PC_C_). In PC_C_ analysis, all images were warped to the average tepal shape using the AAMToolbox program [25, 26]. In this way, instead of comparing the cardinal points, the pixels of each of the appropriate shapes were compared. In total, 30,000 px per image were used based on the RGB (Red, Green, Blue) color model. That is, each image presented 10,000 px and, when multiplied by the three additive primary color components, they gave a total of 30,000 data for the PC_C_.

### Statistical analysis

Principal Component matrices were obtained with the variances of all the main components, both in shape and in color. Of the total components obtained, those that presented modifications in their shape and pigmentation due to the handling of the material were not considered for further analyses. In the end, four components for shape (PC_S_) and four components for coloring (PC_C_) were obtained.

To contrast, the normality of the data, a Shapiro Wilk test was applied. The homogeneity of the variances between the accessions was evaluated with a Levene-test. Because the data did not comply with the assumptions of normality and homoscedasticity, we used non-parametric statistics. Therefore, the significance between the differences in the shape and coloration of the accessions was determined with a Kruskal Wallis test. Finally, to determine the pairs of significantly different groups, a post hoc test (Dunn’s test) was applied on PC_S_ and PC_C_. All these downstream data analyses of PC_S_ and PC_C_ were done using in R (version 2.6.2) [27].

Linear Discrimination Analysis was performed on PCA scores to test the possibility of discriminating the subspecies using morphological shapes and color patterns. Besides the LDA analysis, we also performed a phenetic clustering analysis by using the average PCA values of the accession. We performed all analyses with the complete dataset, including all accession belonging to the subspecies of *M. haageana* and the data set related to the sister species *M. albilanata*.

### Correlation to solar radiation variables

We obtained five environmental variables linked to solar radiation, from the Global Solar Atlas database [28]: GHI, long-term yearly average Global Horizontal Irradiation (kWh/m^2^); DNI, long-term yearly average of Direct Normal Irradiation (kWh/m^2^); DIF, long-term yearly average of Diffuse Horizontal Irradiation (kWh/m^2^); GTI, long-term yearly average of Global Irradiation at Optimum Tilt (kWh/m^2^); and TEMP, long-term yearly average of air Temperature (°C). All of them covered a period of 1999–2018, except for TEMP, which covered a period of 1994–2008. All variables were obtained in *tif* format and adjusted to a 30 arch seconds (approximately 1 km resolution at the Equator). The values were extracted from the occurrence locations, using the packages *raster* ver. 3.4.5 and sp. ver 1.1.4. We used the Pearson coefficient to analyze the correlation between the morphological and solar environmental variables.

## Supplementary Information

**Additional file 1 **Full list of analyzed images, and their corresponding calculated Principal Components for shape (PC_S_) and color (PC_C_). **Additional dataset 1.** Full collection of flattened flowers doi: https://doi.org/10.6084/m9.figshare.14103437.

## Data Availability

All living plant materials are now part of the cacti collection at Jardín Botánico, Instituto de Biología, UNAM. All images used to perform the geometric morphometric analyses were submitted to a public repository doi: 10.6084/m9.figshare.14103437.

## Abbreviations

abE: Abaxial epidermis
adE: Adaxial epidermis
DIF: Long-term yearly average of Diffuse Horizontal Irradiation
DNI: Long-term yearly average of Direct Normal Irradiation
GHI: Long-term yearly average Global Horizontal Irradiation
GTI: Long-term yearly average of Global Irradiation at Optimum Tilt
mc: Mucilage body
LDA: Linear Discriminant Analysis
PCA: Principal Component Analysis
PC_C_: Color Principal Components
PC_S_: Shape Principal Components
RGB: Red, Green, and Blue pixel channels
SD: Standard deviations
subsp.: Subspecies
TEMP: Long-term yearly average of air Temperature

## References

[1] Clegg MT, Durbin ML (2000). Flower color variation: a model for the experimental study of evolution. Proc Natl Acad Sci.

[2] He Q, Shen Y, Wang M, Huang M, Yang R, Zhu S (2011). Natural variation in petal color in *Lycoris longituba* revealed by anthocyanin components. PLoS One.

[3] Bradley D, Xu P, Mohorianu I-I, Whibley A, Field D, Tavares H (2017). Evolution of flower color pattern through selection on regulatory small RNAs. Science..

[4] Vaidya P, McDurmon A, Mattoon E, Keefe M, Carley L, Lee C-R (2018). Ecological causes and consequences of flower color polymorphism in a self-pollinating plant (*Boechera stricta*). New Phytol.

[5] Ho W-C, Ohya Y, Zhang J (2017). Testing the neutral hypothesis of phenotypic evolution. Proc Natl Acad Sci.

[6] Rosas U, Cibrian-Jaramillo A, Ristova D, Banta JA, Gifford ML, Fan AH (2013). Integration of responses within and across *Arabidopsis* natural accessions uncovers loci controlling root systems architecture. Proc Natl Acad Sci.

[7] Rosas U, Barton NH, Copsey L, Barbier de Reuille P, Coen E (2010). Cryptic variation between species and the basis of hybrid performance. PLoS Biol.

[8] Berger BA, Ricigliano VA, Savriama Y, Lim A, Thompson V, Howarth DG (2017). Geometric morphometrics reveals shifts in flower shape symmetry and size following gene knockdown of CYCLOIDEA and ANTHOCYANIDIN SYNTHASE. BMC Plant Biol.

[9] Rosas-Reinhold I, Piñeyro-Nelson A, Rosas U, Arias U (2021). Blurring the boundaries between a branch and flower: potential developmental venues in Cactaceae. Plants..

[10] Mauseth JD (2006). Structure-function relationships in highly modified shoots of cactaceae. Ann Bot.

[11] Bravo-Hollis H. Las cactáceas de México - Tomo I. 2nd ed. Mexico: Instituto de Biología, UNAM; 1978.

[12] Arias S, Guzmán-López L, Guzmán-Cruz U, Vázquez-Benítez B (2012). Cactaceae. Flora del Valle de Tehuacán-Cuicatlán. Fascículo 95.

[13] Cervantes RC, Hinojosa-Alvarez S, Wegier A, Rosas U, Arias S. Evaluating the monophyly of *Mammillaria* series Supertextae (Cactaceae). PhytoKeys. 177:25–42 In press. 10.3897/phytokeys.177.62915 PMC8099837 33967580

[14] Hunt D (1983). A new review of Mammillaria names A-C. Bradleya..

[15] Guzmán U, Arias S, Dávila P (2003). Catálogo de Cactáceas Mexicanas.

[16] González-Sánchez JJ, Santiago-Sandoval I, Lara-González JA, Colchado-López J, Cervantes CR, Vélez P, et al. Growth patterns in seedling roots of the pincushion cactus *Mammillaria* reveal trends of intra- and inter-specific variation. Front Plant Sci. 2021. 10.3389/fpls.2021.750623. 10.3389/fpls.2021.750623 PMC8531529 34691127

[17] Glover BJ, Martin C (1998). The role of petal cell shape and pigmentation in pollination success in *Antirrhinum majus*. Heredity (Edinb).

[18] de Almeida OJG, Sartori-Paoli AA, de Souza LA. Flower morpho-anatomy in *Epiphyllum phyllanthus* (Cactaceae). Rev Mex Biodivers. https://www.biodiversitylibrary.org/part/111950.

[19] Koski MH, Galloway LF (2020). Geographic variation in floral color and reflectance correlates with temperature and colonization history. Front Plant Sci.

[20] Martins C, Oliveira R, Aguiar LMS, Antonini Y (2020). Pollination biology of the endangered columnar cactus *Cipocereus crassisepalus*: a case of close relationship between plant and pollinator. Acta Bot Bras.

[21] Ferreira BH d S, Souza CS, Fachardo ALS, Gomes AC, Sigrist MR (2020). Flowering and pollination ecology of *Cleistocactus baumannii* (Cactaceae) in the Brazilian Chaco: pollinator dependence and floral larceny. Acta Bot Bras.

[22] Martínez-Peralta C, Martínez-Zavala A (2021). Flower biology of the cactus *Coryphantha elephantidens* in the tropical dry forest of Central Mexico. Plant Species Biol.

[23] Guerrero PC, Majure LC, Cornejo-Romero A, Hernández-Hernández T (2018). Phylogenetic relationships and evolutionary trends in the Cactus family. J Hered.

[24] Mosco A (2012). Tissue localization of betacyanins in cactus stems. Rev Mex Biodivers.

[25] Whibley AC, Langlade NB, Andalo C, Hanna AI, Bangham A, Thébaud C (2006). Evolutionary paths underlying flower color variation in *antirrhinum*. Science..

[26] Langlade NB, Feng X, Dransfield T, Copsey L, Hanna AI, Thébaud C (2005). Evolution through genetically controlled allometry space. Proc Natl Acad Sci.

[27] R Core Team. R (2019). A language and environment for statistical computing.

[28] Group WB (2019). Solar resource data obtained from the global solar atlas, owned by the World Bank Group and provided by Solargis.

