# Correction to: Evolution of flower allometry and pigmentation in *Mammillaria haageana* (Cactaceae)

**DOI:** 10.1186/s12870-022-03481-4

**Published:** 2022-03-05

**Authors:** Ulises Rosas, Elisa Sofía Fuentes-Pérez, Cristian R. Cervantes, Estela Sandoval-Zapotitla, Itzel Santiago-Sandoval, Salvador Arias, Jerónimo Reyes-Santiago

**Affiliations:** 1 Jardín Botánico, Instituto de Biología, Universidad Nacional Autónoma de México, 04510 Mexico City, Mexico; 2 Posgrado en Ciencias Biológicas, Universidad Nacional Autónoma de Mexico, Mexico City, Mexico

**Correction to: BMC Plant Biol 22, 52 (2022)**

**https://doi.org/10.1186/s12870-021-03386-8**

Following publication of the original article [1], the authors identified an error in Fig. 4. The correct figure is given below:

The original article has been corrected.

Incorrect Fig. 4

**Fig. 4:**
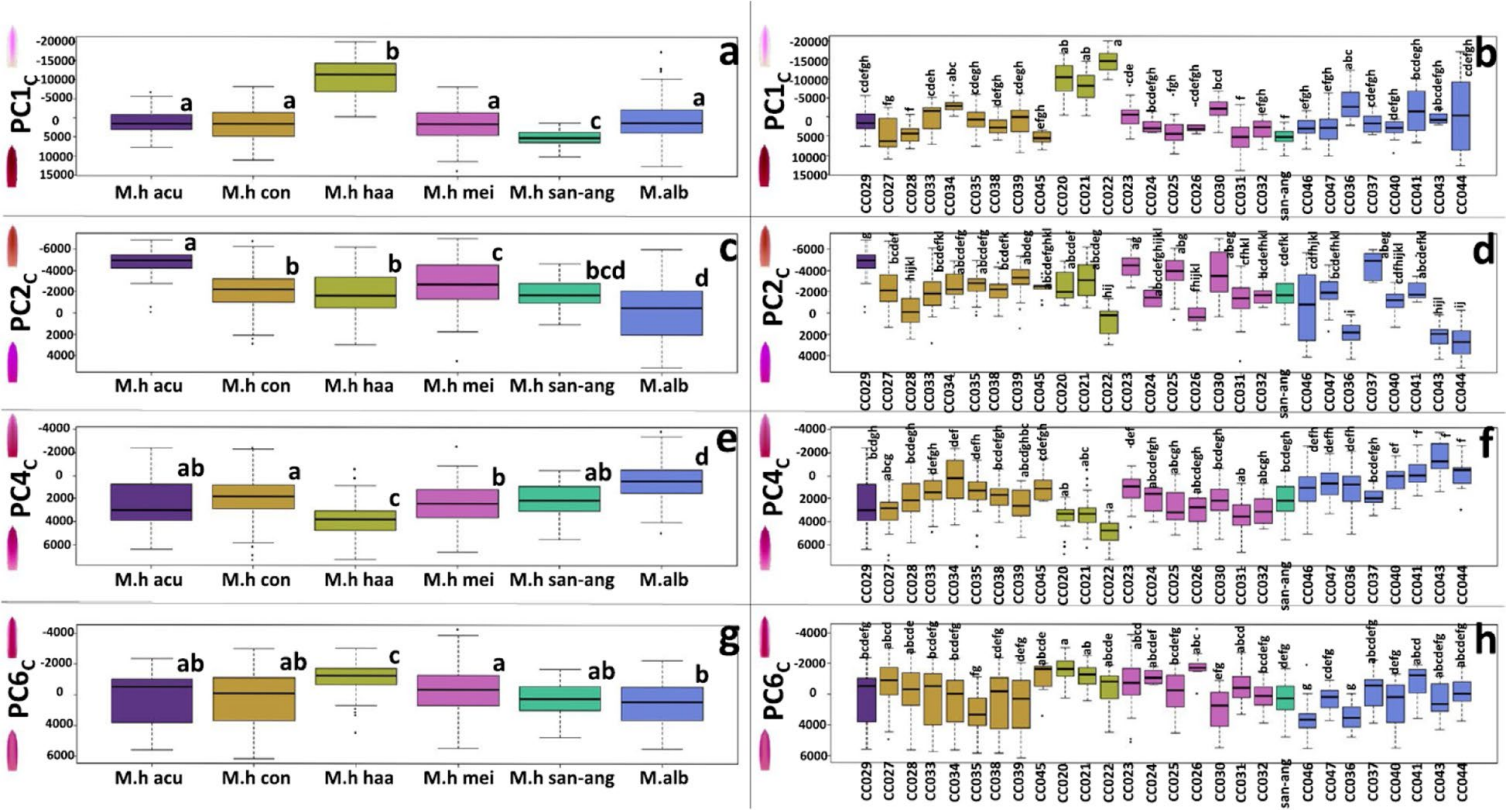
Tepal shape and size variation according to the PCS axes. **a, c, e, g** depicts the PCS groups according to the previously proposed subspecies. **b, d, f, h** depicts the PCS groups according to their corresponding accession. Boxplots show the Q1, Q2, and Q3 quantiles, and outliers. Statistically homogeneous groups from a Dunn test (*p* < 0.05) are also depicted with letters

Correct Fig. 4

**Fig. 4:**
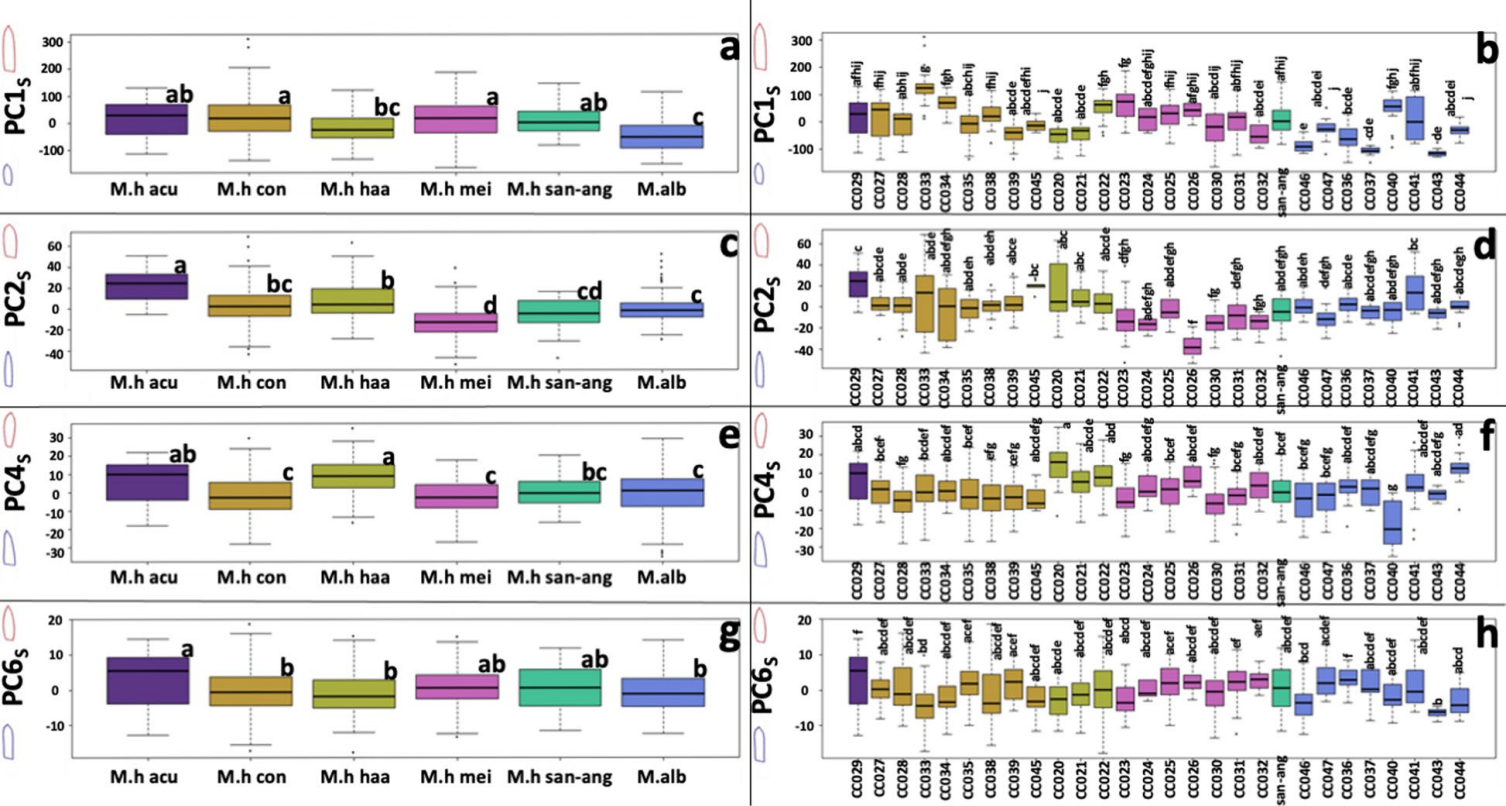
Tepal shape and size variation according to the PCS axes. **a, c, e, g** depicts the PCS groups according to the previously proposed subspecies. **b, d, f, h** depicts the PCS groups according to their corresponding accession. Boxplots show the Q1, Q2, and Q3 quantiles, and outliers. Statistically homogeneous groups from a Dunn test (*p* < 0.05) are also depicted with letters
